# Unveiling the patterns: exploring social and clinical characteristics of frequent mental health visits to the emergency department—a comprehensive systematic review

**DOI:** 10.1007/s44192-024-00070-9

**Published:** 2024-05-27

**Authors:** Zhonghao Zhang, Soumitra Das

**Affiliations:** 1 The University of Melbourne, Melbourne, Australia; 2 Western Health, Footscray, Australia

**Keywords:** Emergency department, Frequent presenter, Frequent presentation, Socio-demographic characteristics, Clinical characteristics

## Abstract

**Background:**

Frequent presenters (FPs) are a group of individuals who visit the hospital emergency department (ED) frequently for urgent care. Many among the group present with the main diagnosis of mental health conditions. This group of individual tend to use ED resources disproportionally and significantly affects overall healthcare outcomes. No previous reviews have examined the profiles of FPs with mental health conditions.

**Aims:**

This study aims to identify the key socio-demographic and clinical characteristics of patients who frequently present to ED with a mental health primary diagnosis by performing a comprehensive systematic review of the existing literature.

**Method:**

PRISMA guideline was used. PubMed, PsycINFO, Scopus and Web of Science (WOS) were searched in May 2023. A manual search on the reference list of included articles was conducted at the same time. Covidence was used to perform extraction and screening, which were completed independently by two authors. Inclusion and exclusion criteria were defined.

**Results:**

The abstracts of 3341 non-duplicate articles were screened, with 40 full texts assessed for eligibility. 20 studies were included from 2004 to 2022 conducted in 6 countries with a total patient number of 25,688 (52% male, 48% female, mean age 40.7 years old). 27% were unemployed, 20% married, 41% homeless, and 17% had tertiary or above education. 44% had a history of substance abuse or alcohol dependence. The top 3 diagnoses are found to be anxiety disorders (44%), depressive disorders (39%) schizophrenia spectrum and other psychotic disorders (33%).

**Conclusion:**

On average, FPs are middle-aged and equally prevalent in both genders. Current data lacks representation for gender-diverse groups. They are significantly associated with high rates of unemployment, homelessness, lower than average education level, and being single. Anxiety disorder, depressive disorder, and schizophrenia spectrum disorders are the most common clinical diagnoses associated with the group.

## Introduction

The hospital emergency department (ED) is the first point of contact when people seek urgent medical care. In Australia, there were 8.8 million ED visits in 2021–2022, with a total population of under 26 million [1]. Other than the well-known physical health emergencies like myocardial infarction or stroke, mental health services are provided in the ED as well. Mental health-related presentations accounted for 3.5% of all ED presentations in the Australian public health system in 2020–2021 [2]. A group of individuals commonly known as the ‘frequent presenter’ (FP) repeatedly present themselves to ED seeking emergency care. Many have labelled this group of patients as ‘frequent flyers’ [3]. They are well known for disproportionally occupying the emergency medical resources and contributing to the increasingly overcrowded ED [4, 5]. They place a significant strain on emergency medical resources, and many frontline healthcare workers have struggled to come up with solutions to reduce the number of presentations [6]. On the other hand, people who present to the ED with mental health complaints tend to face longer waiting hours and poorer care compared to those with general medical conditions [7]. We do acknowledge that this is a complicated issue with a wide variety of different presentations or medical complications within the FP group. Currently, there are gaps in knowledge in terms of the community modelling of the issue, public expectations, and attitudes towards different types of mental health services, and the relationship between FPs’ presentation with their social or clinical factors. To address the situation better, policymakers first need to understand the needs of this group of patients and initiate strategies tailored to their social and clinical characteristics. Numerous studies have been published in the past investigating key features of FPs with mental health conditions. This systemic review aims to identify and consolidate the key socio-demographic and clinical characteristics of FPs with a primary mental health diagnosis from existing literature to provide a starting point for future engagement.

## Method

A comprehensive systematic search and review of published studies on frequent presenters with mental health conditions in hospital ED was conducted based on Preferred Reporting Items for Systematic Reviews and Meta-Analyses (PRISMA) guidelines [8].

Search terms were designed to combine both medical subject headings (MeSH) with common terms used for frequent presenters and/or frequent presentations. 4 databases were accessed for search on 11th of May 2023. MeSH terms were only utilized when searching on PubMed. Table 1 below lists the full search strategy. The term ‘mental health’ was not included in the search strategy in order to include studies on general frequent presentations as well. This is considering that some articles might discuss mental health frequent presentations separately but choose not to include them in the title or abstract.

**Table 1:** Search strategy

Platform	Search strategy	
PubMed	#1	"Emergency Service, Hospital"[Mesh] OR ED OR hospital
	#2	"multiple presentation" OR "multiple attendance" OR "multiple visits" OR "repeated presentation" OR "repeated attendance" OR "repeated visits" OR "frequent presentation" OR "frequent presenter" OR "frequent attendance" OR "frequent representation" OR "frequent visitor" OR "frequent flyer" OR "frequent users" OR "recurrent presentation" OR "recurrent visits" OR "re-presentation"
	#3	#1 AND #2
PsycINFO Scopus WOS	#1	"emergency department" OR ED OR "emergency care" OR hospital
	#2	"multiple presentation" OR "multiple attendance" OR "multiple visits" OR "repeated presentation" OR "repeated attendance" OR "repeated visits" OR "frequent presentation" OR "frequent presenter" OR "frequent attendance" OR "frequent representation" OR "frequent visitor" OR "frequent flyer" OR "frequent users" OR "recurrent presentation" OR "recurrent visits" OR "re-presentation"
	#3	#1 AND #2

The initial search results were exported to Covidence for screening and full-text review

Studies were included if they:

- Were original studies that analysed aspects of trends, patterns, and characteristics of frequent presentations to ED with mental health diagnosis.
- Defined ‘frequent presentation’ as 3 or more ED visits annually or equivalent.
- Were published between Jan 2000 and April 2023 (23 years approximately).
- Were in English.

Studies were excluded if they:

- Were on the paediatric population.
- Were based in military or non-civilian hospital.
- Were reviews or editorials.
- Were not peer-reviewed.
- Did not have full text available.
- Limited the scope of psychiatric conditions.

Articles published to before January 2000 were excluded as the criteria for psychiatric diagnosis, and clinical approach has changed significantly over time. The included time frame generated enough publications for the purpose of this review. Paediatric-focused studies were excluded because many of the socio-demographic characteristics of adults are not applicable to the paediatric population. In addition, frequent presentation in the paediatric population is often psycho-somatic in nature and strongly influenced by family factors [9], which is beyond the scope of this review. The consent process of this cohort lies on their parents or legal guardians, which could jeopardize the accuracy of the data collected.

The following two processes were completed by two authors independently, with conflicts resolved by a third person. After the full text review had been completed, included studies were assessed using the National Heart, Lung, and Blood Institute (NHLBI) Study Quality Assessment Tools [10]. Reference lists of the full-text studies were screened and added for further screening if deemed appropriate. Data extraction was carried out onto a standardized Excel sheet which included: (1) basic study information (primary author, year of publication, country in which the study was conducted, diagnostic criteria); (2) features related to FP (definition of FP, FP number as % of total ED visitor number *vs* number of visits made by FP as % of total visits, sample size, FP admission rate); (3) socio-demographic characteristics (gender, age, employment status, marriage status, homelessness, education level); and (4) mental health conditions (top 3 diagnosis, substance use and alcohol dependence history). After initial data extraction, available data were combined to provide an overview of the social and clinical characteristics of FPs. The studies provided different aspects of the social and clinical characteristics of the patients included. Thus, the overall value was calculated based on corresponding available data only.

## Results

**Fig. 1:**
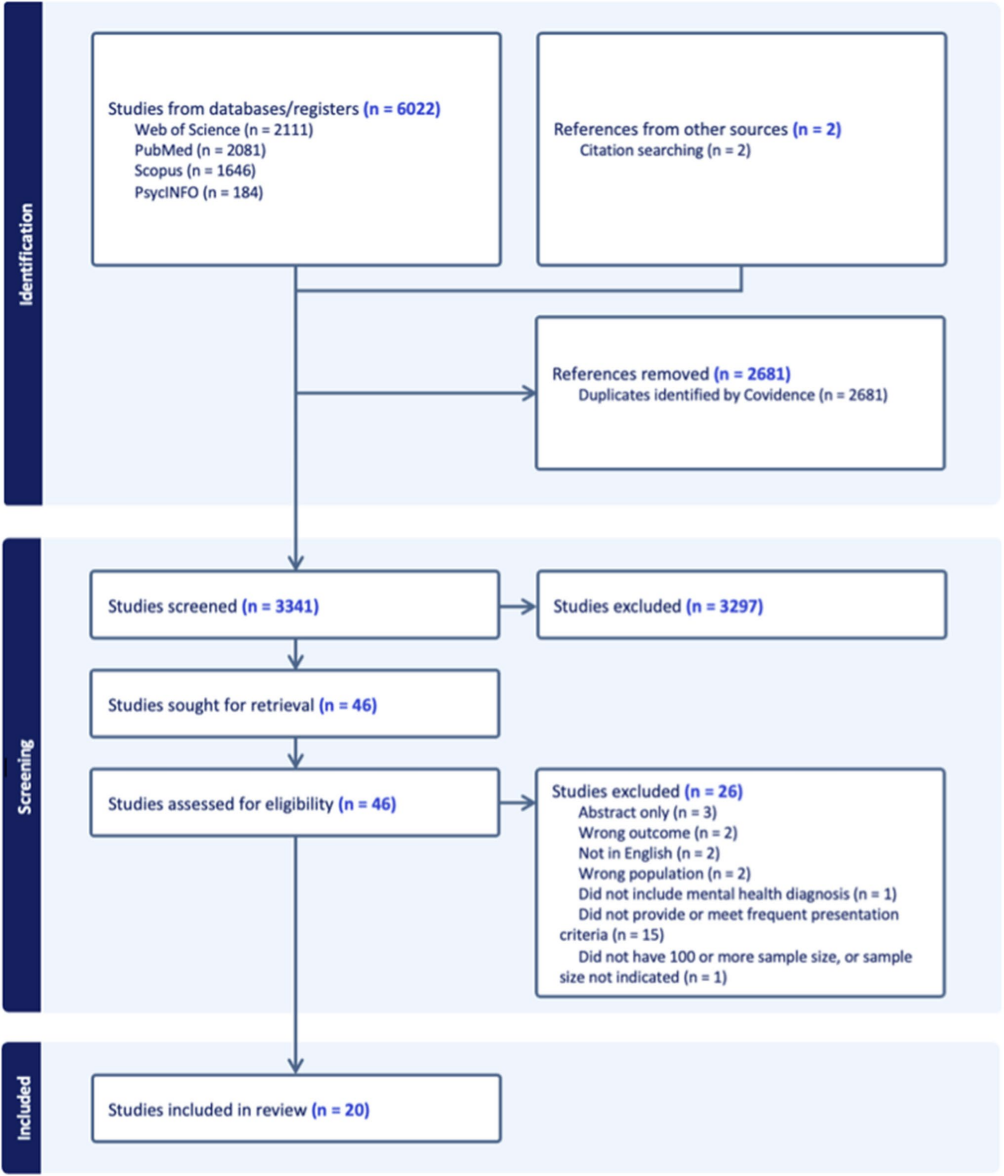
PRISMA

The initial search, as detailed in the Method section, generated 6022 studies, with 2681 identified by Covidence as duplicates. An additional 2 studies were added from citation searching. 3341 studies were screened through title and abstract screening, with 3297 irrelevant studies eliminated. 46 articles 26 were assessed with the full-text review, and 26 were excluded, with the majority reason being that they did not provide or meet frequent presentation criteria (n = 15). The process has been summarised in PRISMA [8] flowchart in Fig. 1. 20 studies were included for the purpose of this review.

All 20 studies can be rated as ‘Good’ based on NHLBI Study Quality Assessment Tools.

Among the 20 studies included published between 2004 and 2022, 6 were from US, 6 from Canada, 4 from Europe and 4 from Australia. 19 studies were retrospective in design and obtained patient data from ED patient record system or equivalent, among which, 1 study [11] combined this data with additional patient interview. Additionally, 1 study [12] is prospective in design and actively recruited patients.

### Sample size and FP definition

Many studies included more than one sample group with different presentation frequencies as a comparison. For the purpose of this review, only the groups that met the inclusion criteria of ‘3 or more times per year or equivalent’ were included in the analysis. The criteria defined in studies that met this criterion varied, ranging from a minimum of ≥ 3 visits/year to a maximum of ≥ 10 visits/year. Many researchers argued that [13] ≥ 4 visits/year should be adopted as universal definition of frequent presentation, with 3 Australian studies included in this review using this as an inclusion criterion. However, multiple recent studies [14–16] with large sample sizes defined frequent presentations as ≥ 3 visits/year. Thus, this definition was chosen to maximize the number of articles and FPs that can be included.

The studies included had sample FP size ranging from 13 to 10,969. Overall, 25,688 FP were included in this review, with mean sample size of 1284. Some studies have demonstrated a significant burden to ED from this group of patients, as demonstrated by FP number as percentage of total ED visitor number (0.1–4.4%) vs. number of total visits made by FP as % of total visits (2.0–8.5%).

### Social profile

19 studies (n = 19) provided the gender of the patients; 52% were male and 48% were female. 1 patient was classified as identifying as neither male nor female. 19 studies included the age of the patients, with some expressed as mean or median and others divided by age group. Notably, there are different ways of assigning age groups, this review has adopted the most commonly used method (≤ 20, 21–40, 41–60 and 60 +). 15% of overall patients have an age 20 and below, 47% fall within 21–40, 25% with age 41–60, and 13% are aged 60 and above. Most studies limited their patient group to aged 12 or 18 and above. 1 study only investigated the young population aged 8–26, omitted from population-wise calculations other than gender and FP admission rate. Overall, the average age was 40.7 years.

As for socio-demographic characteristics, on average, 27% of sampled patients were employed (6–54%, n = 9), 20% married (6–31%, n = 5), 41% homeless (16–78%, n = 8) and 17% have education level as tertiary or above (7–47%, n = 4).

### Clinical profile

19 studies included patients’ diagnoses, with 14 specifying the diagnostic criteria used—either ICD 9/10 or DSM IV/V. Notably, some studies have combined certain common diagnoses (such as anxiety and depression) together. For the purpose of this review, the diagnostic category listed under Table 1 is based on DSM-V [17]. The top three diagnostic categories were summarized from each study, with the final calculation of the top three most diagnosed conditions for frequent presenters being: anxiety disorder (44%), depressive disorders (39%) and schizophrenia spectrum and other psychotic disorders (33%). Other common diagnostic categories include bipolar and related disorders, ‘substance-related and addictive disorders’ and ‘personality disorders. Substance abuse history, including alcohol dependence, was reported by 15 studies, with a prevalence of 44% among frequent presenters.

## Discussion

This review provided an overview of the research conducted on FPs with mental health conditions from 2000 to May 2023. Despite significant differences in terms of sample size, FP definition, and aspects of patient characteristics investigated among studies, this review attempted to assess and summarize social-demographic and clinical characteristics of FPs with mental health conditions on a population basis to be able to inform future development of evidence-based interventions to improve the quality of care and reduce the burden on the healthcare system.

### Profiles of FPs

Most studies have included basic profiles such as gender and age. Overall, there is a slightly higher proportion of male FPs (52%), with 14 studies reporting more males than females in their cohort. On a population basis, despite females higher prevalence of mental health conditions than their male counterparts, female tends to have more internalizing disorders (such as depressive disorders and anxiety disorders), whereas male tends to have more externalizing disorders (including attention-deficit, conduct, and substance use disorder), which could potentially explain the relatively higher frequency of ED visits by male [33, 34]. The FPs typically present with suicidality, substance, or alcohol intoxication with or without psychosis, and frequent relapses of symptoms. Additionally, many presents with significant aggression while in ED, adding to the complicity of managing the FPs. Meanwhile, males are less likely to seek mental health help compared to their female counterparts and more likely to become acutely unwell [35, 36], thus resulting in ED presentations. Multiple factors contribute to this phenomenon, including frequent self-medication with drugs and alcohol and social and certain clinicians’ biases toward masculine stereotypes [37].

The majority of studies only reported the number or percentage of one gender (male or female), with the presumption that gender is a binary concept. Only 1 study [31] has included males and females, females and a third category for gender-diverse groups. This could partially be due to a lack of awareness from researchers and clinicians or a lack of precise and inclusive gender data on existing electronic health record (EHR) systems [38, 39]. However, research has shown that gender-diverse group, in general, reports more mental health conditions than their homosexual and cisgender counterparts [40].

Age-wise, 21–40 years old were identified as the most prevalent age group (47%), with a population average of 40.7 years. These results are reasonable since data has shown that mental and behavioural conditions peak in the 15–34 age group (52.8%) and steadily decline as people get older [34]. Interestingly, the peak and median age at onset for any mental disorder were 14.5 years and 18 years, which might suggest that many symptoms tend to be poorly controlled as people grow to middle age, resulting in frequent presentation [41]. Meanwhile, research in Japan has indicated that people aged 20–39 years tend to have the least help-seeking behaviour, leading to severe psychological distress with potentially acute presentations [42]. As people age, however, there tends to be an increase in self-reported mental health symptoms caused by physical illness as people’s resilience to disease [43].

Given their profile, more mental health campaigns could be aimed at males (MOVEMBER for Men), gender diverse (Rainbow Health Australia) and young adult age groups (Orygen Youth Health in Australia) to promote help-seeking behaviour. Given the young average age of onset, early diagnosis by child and adolescents mental health services with early intervention could potentially reduce the number of future presentations.

### Socio-demographic characteristics

Previous studies have identified income, residence, education, marital and employment status as the principal socio-demographic factors associated with mental disorders [44, 45]. In this review, employment status, homelessness, marital and education status were included for analysis.

The overall employment rate was 27% among the FP group. Unemployment has long been established as more prevalent among people with mental health disorders. According to a report published by OECD in 2012 [46], people with severe mental disorders (SMD) are 6–7 times more likely to be unemployed (45–55%) than people without, and those with common mental disorders (CMD) 2–3 times (30–40%). The OECD data is used as a comparison in this review (where available), considering that all studies included were conducted in OECD countries. Unemployment has been associated with an increase in the probability of poor mental health, with the association most prominent in the long-term unemployment group [46]. Unemployed people commonly report feelings of worthlessness and anhedonia and are more commonly diagnosed with chronic depression and anxiety [47]. However, research is less clear on whether there’s a causal relationship between the two factors, and if there is, which resulted in the other [48, 49].

Financial difficulty as a result of unemployment causes homelessness [50, 51], with 41% of the group recorded as homeless at least once when they presented to ED. Whereas the most recent homeless data reported by OECD countries ranges between 0.01 and 0.86% of the nation’s total population [52]. Among the 6 countries included in this review, despite the US topping the homelessness chart with Switzerland being the lowest, it is not reflective of the population homeless data in either country as both the US and Switzerland's homeless rates were 0.18% in 2020 [52]. On the other hand, Australia has a homeless population of 0.48%, the highest among the 6 countries [52]. Studies have indicated that homelessness is both a cause and a result of mental health conditions [53].

People with a tertiary education level and above was 17%, significantly lower than the OECD average of 31–47% from 2004 to 2021 for 25–34 years-old age group and 18–30% in the same period for 55–64 years-old [54]. Studies have shown that better education helps with better access to psychosocial resources, such as a sense of control, resilience, and cultural activities, which all contribute to good mental health [55].

Marital status is another major socio-demographic factor. On a population basis, the number of adults ages 25 to 54 who are married fell from 67% in 1990 to 53% in 2019 in the US [56], which is still significantly higher than the FP group’s marriage rate of 20%. Studies have found that marriage could provide psychological benefits and is a protective factor for mental health [57–59]. However, it can be contradictory as sometimes unstable marriage and early marriage can increase distress [60].

From the data above, we can argue that by addressing the existing social challenges such as unemployment, homelessness, poverty, and low education levels, the government is also tackling and reducing the potential mental health crisis experienced by the targeted group of individuals. More mental health education could also be provided at schools, universities, and workplaces to promote awareness. Social welfare institutes could liaise with community mental health teams to provide counselling services for those without employment or home.

### Clinical characteristics

Statistically, it is unsurprising that anxiety disorder (44%) and depressive disorders (39%) top the diagnostic charts as they are also the first and second most prevalent mental health conditions in the US, with population annual prevalence of 19.1% and 8.4% respectively [61]. However, schizophrenia spectrum and other psychotic disorders, which represent 33% of frequent presentations, have much lower population prevalence, between 0.25% and 0.64% [61]. It is unsurprising that schizophrenia represents a tiny portion of the population with mental health conditions that present so often. Schizophrenia can present with a wide range of positive and negative symptoms [62], such as hallucinations, delusions, and disturbances in thought and behaviour. Meanwhile, 40% of schizophrenia patients are also affected by depression, 65% affected by anxiety symptoms [63, 64]. All of these contribute to the high presentation rate in this group of patients despite representing a small portion of psychiatry diagnoses in the community. The presentation rate is lower than depression and anxiety, which could be explained by the established community-based care models for schizophrenia that partially reduce the emergency presentations to hospitals [65]. Citing the high prevalence of depression and anxiety in both the community and among the FP group, we urgently need a community-based model for their treatment and management. More training could be provided for general practitioners (GPs) to improve their knowledge of psychiatry.

In many cases, those who presented to ED were brought in by law enforcement agencies against their free will due to patients’ lack of insight [66]. Some presentations of schizophrenia, such as acute psychosis, require timely hospital treatment with multidisciplinary input, which has been associated with lower rehospitalization rates and improved psycho-social skills [67]. It has been well-established that early diagnosis and intervention bring better outcomes [68]. However, developed countries generate much more research in the field than underdeveloped regions; thus, the general consensus might be biased and new studies with new diagnostic criteria and intervention plans might generate different results.

Another frequent diagnosis of FPs reported by frontline ED staff is borderline personality disorder (BPD). However, only 3 studies have identified personality disorder as the top 3 diagnoses. A Spanish study [69] has identified that 9% of total visits to psychiatric emergency services involved a diagnosis of BPD, among which 63% were female, 33% had depression or anxiety, 45% with substance use disorder, and 28% presented with disruptive behaviour. A study in Australia [70] has found similar results, with 15% of BPD patients having concurrent schizophrenia or mood disorder. However, on hospital records, BPD patients only represent less than 2% of total ED presentations in general hospitals, despite the perception that people with BPD are disproportionately reliant on emergency services [70]. A study has found that many psychiatrists are not willing to disclose or document the diagnosis of BPD due to stigma and uncertainty [71]. Meanwhile, frontline ED staff have less training in this field than mental health specialists and might not have enough time to explore the diagnosis. In addition, BPD patients usually present with a diagnosis that is more severe and easier to diagnose, such as the abovementioned substance use disorder, depression, anxiety, and schizophrenia, which are commonly documented. Substance abuse, including alcohol dependence, is widely known to be more prominent in people with mental health conditions. An estimated 2% of the world's population is dependent on alcohol or an illicit drug [80], with a lifetime prevalence of 6.5% for alcohol and 8.9% for substances [81], much lower than the 44% from the FP cohort. 7 studies have included substance-related and addictive disorders as their top 3 diagnoses. Over half of patients with substance and alcohol dependence are associated with co-occurring mental health conditions such as mood disorder, depression and anxiety, and schizophrenia [72, 73], which further contributed to their ED visits on top of their acute toxication episodes.

Bipolar and related mood disorders (BD) are another commonly seen diagnosis in FPs. It is the top 3 diagnosis in 7 out of 20 studies included in this review. More than 2.2 million ED visits were made by patients with BD in the US in 2018, among which 300 thousand patients received BD as the principal diagnosis, with most others present with suicidal ideations, anxiety disorders or substance-related concerns [74]. Unsurprisingly, many of the BD patients come from lower-income households [74]. Another study in Latin America in 2011 [85] also mentioned that BD patients were more likely to report alcohol abuse, ADHD, depression, and panic disorders with significantly higher suicidal ideation than non-BD patients, which potentially predisposes to their frequent presentation.

Given FP’s clinical profile, all of the top 3 conditions identified are manageable in community to prevent acute relapse thus reducing number of hospital presentations. Early diagnosis, early intervention, and continuous follow-up with safety-netting are the keys to keep them away from ED.

### Influence on care

Many studies have acknowledged the strain on the medical workforce from the FP group with mental health conditions. As illustrated in Table 2, this group of patients tend to use ED resources disproportionally more (as demonstrated by ‘FP number as % of total ED visitor number *vs* number of visits made by FP as % of total visits’) compared to the general population. Despite frequent presentations, their overall admission rate of 26% is very similar to that of the general population—28% in Australia [1], and 20% in the US [75]. Often, people have longer stay in the ED, causing obstacles for other patients in genuine need to come for help. In Australian settings, 10% of ED mental health presentations had a length of stay longer than 16 h in 2020–2021, much longer than the same measure for all ED presentations of 9 h [2]. The Australian Medical Association (AMA) reported in 2022 that those with mental health admission requirements wait, on average of up to 28 h before admission due to a lack of hospital beds [76]. Some visits could be easily prevented with community support such as GPs, mental health nurses, peer workers or family support. People with mental health distress often find it hard to navigate complex community resources, so they seek ED care where 24-h care is available.

**Table 2:** Characteristics of included studies

Primary author	Publication year	Country	Diagnostic criteria	FP number as % of total ED visitor number *vs* number of visits made by FP as % of total visits	FP definition	Sample FP size	FP admission rate	Socio-demographic characteristics n (%)												Top 3 diagnosis: category (%)
								Gender			Age				Employment status	Marriage status	Homelessness	Education level	Substance abuse history*	
								Male	Female	Others	≤ 20	21–40	41–60	60 +	Employed	Married	Yes	Above secondary	Yes	
Arfken [12]	2004	US	NA	NA	≥ 6v/year	74	30%	74%	26%	NA	Mean 40.6				NA	NA	60%	NA	35%	2(70%), 8(35%)
Pasic [18]	2005	US	ICD-10	4.4% vs NA	≥ 4v/qr	204	NA	74%	26%	NA	Mean 36.9				45%	NA	78%	NA	34%	8(22%), 2(15%), 4&5(15%)
Ledoux [19]	2005	Belgium	DSM-IV	NA	≥ 4v/16 m	100	NA	70%	30%	NA	Mean 33.6				19%	NA	NA	NA	NA	NA
Brunero [20]	2007	Australia	ICD-9	1.5% vs 5.6%	≥ 4v/year	13	46%	62%	38%	NA	8%	62%	30%	0	NA	NA	NA	NA	NA	4&5(39%), 2(15%)
											Mean 33									
Mehl-Madrona [11]	2008	US	DSM-III-R	NA	≥ 6v/year	200	NA	41%	59%	NA	18%	34%	21%	27%	NA	NA	NA	NA	31%	4(24%), 8(19%), 5(16%)
Wooden [21]	2009	Australia	ICD-10	0.47% vs 4.5%	≥ 1v/m	54	NA	NA			Mean 42.8				NA	NA	NA	NA	NA	9(33%), 2(25%), 4&5(23%)
Vandyk [22]	2014	Canada	NA	0.1% vs 3%	≥ 5v/year	70	NA	66%	34%	NA	60%		34%	6%	19%	9%	24%	NA	67%	2(30%), 3(20%), 8(20%)
Brennan [23]	2014	US	ICD-9	3.3% vs 8.5%	≥ 4v/year	2394	NA	57%	43%	NA	NA				NA	NA	NA	NA	26%	2(56%), 3(54%), 5(48%)
Chang [24]	2014	US	NA	NA	≥ 4v/year & ≥ 3v/2 m	167	NA	56%	44%	NA	37%		53%	11%	NA	31%	23%	NA	53%	3(69%), 8(46%), 5(23%)
Vu [25]	2015	Switzerland	NA	0.9% vs NA	≥ 5v/year	220	NA	55%	45%	NA	Mean 51.5				54%	NA	NA	19%	61%	8(61%), 4(47%), 5(34%)
Richard-Lepouriel [26]	2015	Switzerland	DSM-IV	NA	≥ 3v/year	210	NA	49%	51%	NA	Mean 38.7				16%	25%	NA	NA	52%	8(52%), 3(49%), 9(40%)
Sirotich [27]	2015	Canada	NA	NA	≥ 2v/6 m	146	NA	43%	57%	NA	Mean 42.1				18%	NA	18%	NA	29%	3(52%), 2(30%), 8(39%)
Buhumaid [28]	2015	US	DSM-IV	0.27% vs 2.0%	≥ 4v/year	126	22%	64%	36%	NA	33%		67%		NA	NA	37%	NA	81%	2(63%), 4(42%), 3(15%)
											Mean 43.5									
Meng [29]	2017	Canada	ICD-10	NA	≥ 10v/year	34	NA	65%	35%	NA	Mean 40				15%	NA	50%	21%	65%	8(53%), 5(26%), 4(12%)
Fleury [14]	2019	Canada	ICD-9/10	NA	≥ 3v/year	10,969	NA	53%	47%	NA	NA				NA	NA	NA	NA	46%	5(50%), 4(45%), 2(34%)
Slankamenac [30]	2020	Switzerland	NA	NA	≥ 4v/year	45	NA	62%	38%	NA	Mean 42.7				13%	28%	16%	47%	36%	8(44%), 9(22%), 2(11%)
Casey [31]	2021	Australia	ICD-10	NA	≥ 4v/year	200	NA	53%	46.5%	0.5%	13%	58%	28%	2%	6%	6%	NA	7%	NA	9(15%), 2(14%), 3(9%)
											Mean 33.4									
Gentil [15]	2021	Canada	ICD-9/10	NA	≥ 3v/year	3121	NA	54%	46%	NA	NA				NA	NA	NA	NA	41%	5(56%), 7(43%), 4(48%)
Armoon [16]	2022	Canada	ICD-9/10	NA	≥ 3v/year	5510	NA	47%	53%	NA	NA				NA	NA	NA	NA	48%	4&5(85%), 2&3(57%)
Cullen [32]	2022	Australia	ICD-10	NA	≥ 4v/year	1831	41%	43%	57%	NA	Range 8–26 only				NA	NA	NA	NA	NA	5(15%), 4(9%)
Combined Data: calculated with corresponding available data only						25,688	26%	52%	48%	NA	15%	47%	25%	13%	27%	20%	41%	17%	44%	5(44%), 4(39%), 2(33%)
											52%		48%							
											Mean 40.7									

Category based on DSM-V [17]:

1: Neurodevelopmental Disorders

2: Schizophrenia Spectrum and Other Psychotic Disorders

3: Bipolar and Related Disorders

4: Depressive Disorders

5: Anxiety Disorders

6: Obsessive-Compulsive and Related Disorders

7: Trauma- and Stressor-Related Disorders

8: Substance-Related and Addictive Disorders

9: Personality Disorders

10: Other diagnosis listed under DSM-5

FP: frequent presenter; m: month; qr: quarter; yr: year; v: visits; NA: not mentioned in the study or not applicable

^*^Including alcohol dependence, if available

We acknowledge that there are many forms of bias and social stigma on people with mental health conditions, especially in less developed countries. A 2016 report has indicated that mental disorders are subject to the greatest amount and extent of negative judgements and stigmatization, which have long-lasting and devastating effects on patients’ illnesses [77]. Social exclusion and prejudices could also exacerbate their symptoms of mental illness [77]. As a result, many mental health conditions are not diagnosed or treated in their early stages, thus resulting in hospital ED presentation during the acute episode. For FPs, on top of the bias experienced in society, many frontline ED staff have labelled the FP group as ‘frequent flyers’ out of frustration, which partially stems from the lack of management knowledge despite recognizing them as being vulnerable [6]. From the patients’ perspective, many have complained of a lack of empathy from the treating team and felt unappreciated or misunderstood [78]. From the medical system’s perspective, difficulties in resolving the current situation create a ‘revolving-door’ service circle [79], thus resulting in more presentations and frustration from both sides of the medical service, in addition to the financial cost of public medical insurance. Meanwhile, in full-service hospitals, critical emergency resources could be drained away by community-manageable mental health presentations from more urgent conditions such as myocardial infarctions or strokes, which might result in an overall reduction in healthcare outcomes. It is worthwhile to mention that there is a need for a new study to be conducted in Australia on FP to know if there is a change in trend and community perception around the issue and to know how the stigma from ED staff on mental health FPs has changed the presentation pattern over time.

Clearly, there is a significant gap between the demand of community mental health services needed by the FPs and the model or level of care currently provided. This will be addressed under ‘Suggestions’.

### Suggestions

Before we dive into strategies to tackle the current crisis, we recognize the importance of patients present to the ED with the appearance of mental health conditions also to be assessed by general ED physicians to rule out general medical causes of mental health issues, such as delirium or thyroid conditions. Many models to address the FP issue have been proposed in the past [80]. General mental health presentations are a result of acute stressors on top of their long-term socio-demographic disadvantage and clinical conditions. Dedicated mental health triage services and emergency psychiatrists can help reduce bed-block and reserve critical medical resources for patients with general medical conditions. ED staff training can include basic knowledge of dealing with frequently presenting patients, thus reducing frustration. Interventions with inter-professional approach has proven beneficial in FP’s care, with reduction in ED presentation frequencies [81]. For FP with mental health conditions, the multi-disciplinary treating team can include the patient’s treating psychiatrist, psychologist, mental health nurse, social worker, and other healthcare professionals relevant to the patient’s condition.

Despite the importance of reducing ED waiting times and more hospital beds for mental health patients, we strongly advocate for more resources to be allocated to community mental health services with multiple benefits. On a community level, case-based management and a patient-centred approach can be tailored to specific patients’ needs, with a higher rate of patient satisfaction, better clinical outcomes, and reduced ED visits and admissions [82, 83]. Nursing models such as the Buurtzorg [84] could be expanded to provide mental health support and social welfare services at a local level. In Australia, the Royal Commission on Victoria’s Mental Health System [85] (the Commission) has recommended integrating mental health and wellbeing services into local communities with a regionalised governance body.

In case of a mental health crisis, the Commission recommended 24-h telehealth services that provide triage and emergency support. Such services have been made available in many developed countries like Australia (Lifeline, Beyond Blue), Canada (Talk Suicide Canada), and the US (988 Suicide & Crisis Lifeline) etc., with most being 24/7 and toll-free. However, many developing countries still lack dedicated suicide prevention services staffed by mental health professionals. Many authorities refer people at crisis to emergency police lines, which are not necessarily staffed by people good at mental health crisis management. Such hotline services could be complemented by outreach services from peer workers on site and followed up by mental health professionals. Community wellbeing centres and domestic violence services can provide safe spaces and crisis respite facilities, which play the role of hospital ED to reduce unnecessary presentations, thus improving the overall quality of care in ED. Social housing programs, financial assistance for the unemployed, and better education infrastructure could reduce the incidence of mental health conditions by addressing the identified risk factors for frequent presentations. Involuntary treatment orders should always be considered as a measure of last resort, with thorough police training delivered by clinicians. Mental health services and law enforcement agencies could also engage in community education on related regulations to promote awareness.

Funding is the key to all policies and medical treatment. An OECD report from 2021 indicated that mental ill-health drives economic costs of more than 4% of countries GDP [86]. The report has praised countries like Australia, Canada, New Zealand, Norway and the UK for leading the mental health campaign [86]. However, despite the growing attention, OECD countries, in general, have not significantly increased funding for mental health in the past [86]. Given the Australian example, government spending on mental health-related services increased by an average rate of 4% annually between 2016–2017 and 2020–2021, with an average annual increase of only 2.8% in real terms [87]. This has led to the AMA statement in 2018 claiming that ‘mental health and psychiatric care is grossly underfunded when compared to physical health’ in Australia [88]. A similar situation is seen in Canada, where the Canadian Mental Health Association criticized the nation’s Budget 2023 being ‘out of touch with mental health crisis’ [89]. In addition, as identified by this review, 44% of the FPs with mental health conditions have a history of substance abuse. Resources for addiction treatment could also be another area of major investment to consider by the future government budget.

### Limitations and implications on future research

There are several limitations to this review. First, only English publications with available full texts were included (conference abstracts without full texts articles published in English were excluded), which could lead to language and accessibility bias. Second, as mentioned above, few studies have included gender-diverse grouping in their patient profile. Given the prevalence of mental health issues among the group [40], future studies and electronic health records should take this into account. Third, there are significant differences in frequent presentation definitions among different studies. Most studies have defined frequent presentation as 3 or 4 more visits per year, with some others defining the criteria on a monthly basis. As such, patients with different presentation frequencies were analysed together in this review. However, those who present 10 times a year more may have a different social or clinical profile than those who present 4 times a year. Standardized approach to define frequent presentations should be adopted in the future. In addition, studies sampled limited and different aspects of the socio-demographic or clinical profiles of their respective patient cohort. This review only included the most relevant aspects, but there are many others worth analysing, such as patients’ source of referral, primary care usage, and past admission history. Moreover, most studies did not include the acute stressors that prompt patients’ ED visits or their utilization of community resources such as general practitioners or private psychiatrists. Future studies could include as many aspects of patients’ profiles as possible. This could be helpful in identifying the immediate need and predisposing factor of their presentations. Different diagnostic tools used also add to the difficulties in standardized analysis. A gold standard and regulated version of mental health diagnostic tools should be developed and implemented. The goal of these strategies is to facilitate mental health care on a community level to reduce the number of presentations, thus promoting the overall healthcare outcome.

## Conclusion

Frequent presenters with mental health condition utilize the emergency medical services disproportionally compared to the general public. This review is one of the first studies that summarized the profile, socio-demographic and clinical characteristics of FPs with mental health conditions. On average, FPs are middle aged and equally prevalent in both gender. Current data lacks representation for gender diverse group. They are significantly associated with high rate of unemployment, homelessness, lower than average education level, and being single. Anxiety disorder, depressive disorder, and schizophrenia spectrum disorders are the most common clinical diagnosis associated with the group. However, we also acknowledge the limitations of this review, stemming from the exclusion of non-English articles, difference in frequent presentation definition and a lack of standardized approach to define and analyse this unique group of patients. Based on our findings, we have made recommendations to improve care for this group of patients, including ED staff training, better community mental health resourcing, 24/7 telehealth hotline service, and stronger government funding on mental health. This review serves as starting point for those looking to develop a care model addressing the frequent presentation issue and improving the overall health outcomes from emergency department utilization.

## Data Availability

No datasets were generated or analysed during the current study.

## Abbreviations

AMA: Australian Medical Association
BD: Bipolar disorder
CMD: Common mental disorders
DSM: Diagnostic and statistical manual of mental disorders
ED: Emergency Department
EHR: Electronic health record
FPs: Frequent presenters
ICD: International classification of diseases
MeSH: Medical subject headings
NHLBI: National Heart, Lung and Blood Institute
OECD: Organization for Economic Co-operation and Development
PRISMA: Preferred Reporting Items for Systematic Reviews and Meta-Analyses
SES: Socio-economic status
SMD: Severe mental disorders
WOS: Web of science
